# Driving distances and loss to follow‐up after hematopoietic cell transplantation

**DOI:** 10.1002/jha2.179

**Published:** 2021-03-04

**Authors:** Rahul Banerjee, Alison W. Loren

**Affiliations:** ^1^ Division of Hematology/Oncology Department of Medicine University of California San Francisco San Francisco California USA; ^2^ Division of Hematology/Oncology Perelman School of Medicine University of Pennsylvania Philadelphia Pennsylvania USA

**Keywords:** geographic factors, hematopoietic cell transplantation, lost to follow‐up, telemedicine

## Abstract

In a recent multicenter analysis, long geographic distances predicted loss to follow‐up (LTF) among allogeneic hematopoietic cell transplantation (HCT) survivors. We hypothesized that lower frequencies of patient interactions (including in‐person appointments and telemedicine encounters) would predict LTF rather than long driving distances. However, in our retrospective single‐center analysis of 263 HCT survivors, the only predictors of LTF were residence in the furthest driving‐distance quartile and Medicaid insurance (but not annualized frequencies of patient interactions). Our findings suggest that telemedicine may not necessarily "rescue" long‐distance HCT survivors from LTF. Other solutions, for example patient‐specific partnerships with local providers, may be helpful.

## 1 INTRODUCTION

Allogeneic hematopoietic cell transplantation (HCT) for hematologic malignancies requires long‐term follow‐up for chronic graft‐versus‐host disease (cGVHD) management and optimal survivorship care. However, in a recent Center for International Blood and Marrow Transplant Research (CIBMTR) analysis, loss to follow‐up (LTF) rates among allogeneic HCT survivors rose to 5% within 5 years of HCT and 13% within 10 years of HCT [1]. Long geographic distances between patient residences and HCT centers predicted LTF in this analysis; however, data on appointment frequencies were not available for incorporation into CIBMTR regression modeling. Previous research suggests that long‐distance HCT survivors may attend fewer in‐person appointments compared to nearer‐living patients [2]. Additionally, because telemedicine‐based follow‐up appointments are a recommended component of post‐HCT care for long‐distance survivors [3], it is unknown whether this observed reduction in attended in‐person appointments is offset by a compensatory rise in telemedicine encounters among long‐distance patients. We hypothesized that lower frequencies of *patient interactions*, a term including both in‐person appointments and telemedicine encounters, would independently predict LTF among HCT survivors rather than long driving distances.

## 2 METHODS

We conducted a retrospective landmark analysis of adult allogeneic HCT survivors alive 1+ year after HCT seen at a single academic center in the United States that historically performs 50–100 allogeneic HCTs per year. Data were collected between January 1, 2002 and June 30, 2018. To mirror CIBMTR classifications at this stage of survivorship, we defined LTF as the absence of follow‐up for 2+ years [1]. However, we defined follow‐up more broadly to include any form of patient interaction; this term included both in‐person appointments and telemedicine encounters (as delineated based upon encounter codes entered by clinics). In‐person appointments included clinician visits, infusion visits, and appointments with allied healthcare providers such as social workers or physical therapists. Telemedicine encounters included virtual visits, scheduled clinic‐initiated phone calls, and electronic clinic‐initiated messages sent via email or patient portals. As done previously, we classified HCT survivors into furthest‐quartile (Q4) or nearer‐quartiles (Q1‐3) groups based on the 75th percentile of driving distance [4]. For each HCT recipient, we totaled and annualized interactions between Day +366 and last known follow‐up. We compared the Q4 and Q1–3 groups using Fisher's exact testing and Wilcoxon rank‐sum testing with *p* < 0.05 to signify statistical significance. We used logistic regression modeling with backward stepwise elimination (eliminating for *p* > 0.05) to assess the impacts of dichotomous driving distance group (Q4 versus Q1–3), patient interaction frequencies, and other covariates (pre‐HCT diagnosis, age at HCT, race, gender, insurance status, HCT done at index versus separate center, days of follow‐up after HCT, and presence of cGVHD at any point) on LTF.

## 3 RESULTS

We analyzed 263 HCT recipients as described in Table 1. The median year of HCT was 2013 with interquartile range (IQR) 2009–2015. Median follow‐up was 49 months after HCT (IQR 28–93 months). Driving distances ranged from 2 to 1063 miles with a median distance of 32 miles and IQR 20—59 miles; the Q4 group (*n *= 65) thus comprised patients with driving distances of 59 miles or higher from the index center. Among all patients, a median of 20 interactions occurred per year of follow‐up (IQR 10–44 interactions per year). The Q4 and Q1–3 groups were similar with regard to pre‐HCT diagnosis, age, gender, insurance status, HCT done at index versus separate center, and presence of cGVHD. However, the Q4 group was more likely to be white. Median follow‐up intervals for the Q1–3 and Q4 groups were comparable (51 months versus 49 months, *p* = 0.30). Of 27,347 analyzed interactions, 1370 (5%) were telemedicine encounters. The proportion of all interactions that were telemedicine encounters were statistically different but clinically similar between the Q1–3 and Q4 groups (5% vs. 7%, *p* < 0.01).

**TABLE 1:** Characteristics of patients based upon driving distances

	**Q1‐3**	**Q4**	**Total**	***p* value**
**Overall**	198 (75%)	65 (25%)	263 (100%)	
**Pre‐HCT diagnosis**				0.41
Acute leukemia	153 (77%)	47 (72%)	200 (76%)	
Other condition	45 (23%)	18 (28%)	63 (24%)	
**Age at HCT**				0.58
≤40 years	59 (30%)	24 (37%)	83 (32%)	
41–64 years	114 (58%)	34 (52%)	148 (56%)	
≥ 65 years	25 (13%)	7 (11%)	32 (12%)	
**Race** (missing for *n* = 6)				0.03*
White	169 (87%)	61 (97%)	230 (87%)	
Black	16 (8%)	0 (0%)	16 (6%)	
Other	9 (5%)	2 (3%)	17 (6%)	
**Gender**				1.00
Male	95 (48%)	31 (48%)	126 (48%)	
Female	103 (52%)	34 (52%)	137 (52%)	
**Insurance status** (missing for *n* = 3)				0.75
Commercial	155 (79%)	51 (79%)	206 (79%)	
Medicaid	7 (4%)	1 (2%)	8 (3%)	
Medicare	33 (17%)	13 (20%)	46 (18%)	
**HCT center**				1.00
Index center	184 (93%)	61 (94%)	245 (93%)	
Different center	14 (7%)	4 (6%)	18 (7%)	
**cGVHD**				0.67
Present at any point	93 (48%)	28 (44%)	121 (47%)	
Absent	102 (52%)	35 (56%)	137 (53%)	

Patients were divided into Q1–3 versus Q4 groups based on the 75th percentile of driving distance.

^*^Significant at *p* < 0.05.

Abbreviations: cGVHD, chronic graft‐versus‐host disease.; HCT, hematopoietic cell transplantation.

**FIGURE 1:**
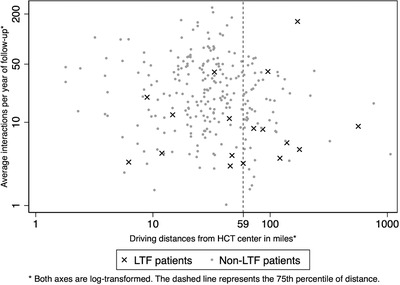
Scatter plot of interaction frequencies versus driving distances among HCT survivors alive 1+ year after HCT. Abbreviations: HCT, hematopoietic cell transplantation; LTF, loss to follow‐up

Overall, 17 patients (6% of our cohort) were found to be LTF. As depicted in Figure 1, the Q4 group comprised almost half (47%) of LTF patients; however, the correlation between interaction frequencies and driving distances was weak (Pearson's coefficient −0.10). The Q4 group had lower interaction frequencies (medians 14 vs. 23 interactions per year, *p* < 0.01) and higher LTF rates (12% [8/65] vs. 5% [9/198], *p* = 0.03) compared to the Q1–3 group. Among Q4 patients, interaction frequencies were lower among LTF patients (median 8 vs. 17 interactions per year of follow‐up) but this did not reach significance (*p* = 0.29). After performing logistic regression with backward stepwise elimination, the only predictors of LTF in our final model were Q4 driving‐distance status (odds ratio [OR] 4.06, 95% confidence interval [CI] 1.34–12.2, *p* = 0.01) and Medicaid insurance (OR 9.38, 95% CI 1.50–58.5, *p* = 0.02).

## 4 DISCUSSION

Contrary to our initial hypothesis, we found long driving distance to be a superior predictor of LTF among HCT survivors compared to annualized interaction frequencies (encompassing both in‐person appointments and telemedicine encounters). Compared to a recent multicenter CIBMTR analysis demonstrating a 5% rate of LTF 5 years after allogeneic HCT [1], our corresponding 6% LTF rate with median follow‐up of 49 months was similar. Additionally, our findings were consistent with the CIBMTR's models, suggesting that driving distances of 100+ miles and public insurance status were associated with LTF. While we defined long driving distances based on the 75th percentile rather than a predefined cutoff of 100+ miles as used by the CIBMTR, these definitions were analogous given that 100 miles constituted the 71st percentile of driving distance in the CIBMTR analysis [1]. As such, our research builds upon this previous analysis by demonstrating that long driving distances and Medicaid insurance retain their association with higher rates of LTF even after accounting for annualized interaction frequencies and cGVHD (data about which were not available to the CIBMTR).

Limitations of our retrospective cross‐sectional analysis include its relatively small sample size from a single institution, because of which we were only able to identify 17 LTF patients. Given that 7% of patients in our cohort had originally undergone HCT at a different center (similar to the proportion at another academic US center) [4], it is possible that LTF patients may actually have transitioned their follow‐up to a different HCT center for geographic or personal reasons. A final limitation of our retrospective analysis is the low usage of clinic‐initiated telemedicine encounters, which comprised only 5% of interactions in our analysis of data from 2002 to 2018. Of note, this figure may underestimate the global usage of telemedicine in this population because our methods did not allow us to capture telephone calls or electronic messages that were initiated by HCT survivors rather than by a clinical provider. On a more general level, telemedicine encounters among HCT survivors are likely more prevalent in recent years given increasing familiarity with digital technologies as well as the social distancing requirements created by the coronavirus disease 2019 (COVID‐19) pandemic.

Our findings nevertheless highlight a potential limitation of previous studies that have investigated driving distances and outcomes in malignant hematology. These studies have generally found no impact of long distances on either clinical or patient‐reported outcomes [2, 4, 5, 6, 7. If certain long‐distance patients are disproportionately both at risk of LTF and worsened outcomes, retrospective analyses with censoring may fail to capture the entirety of their post‐HCT courses. From a clinical perspective, our data also suggest that telemedicine itself may not necessarily "rescue" long‐distance HCT survivors from higher LTF rates. Other solutions to coordinate care, for example proactive patient‐specific partnerships with local providers using direct email/phone communication channels [3, 8, may be helpful components of care for long‐distance survivors in addition to scheduled telemedicine encounters. Tailored strategies to bridge the "digital divide" among patients who have less familiarity with videoconferencing technology—for example, dedicated personnel available in advance or on demand (via telephone) to troubleshoot technical issues—may enhance the acceptability of telemedicine for underserved patients with cancer [9, 10. Lastly, because HCT survivors may prefer paper copies of their survivorship care plans over web‐based attachments [11], important follow‐up information after telemedicine encounters can potentially be mailed to patients at potential risk of LTF as well.

## 5 CONCLUSION

We found that long driving distances among HCT survivors are associated with higher rates of LTF even after adjusting for how frequently these patients interact in person or virtually with their HCT center. Mitigating the risk of LTF among long‐distance survivors may require a multipronged approach with both telemedicine adoption as well as other personalized strategies to maintain engagement among these vulnerable patients.

## CONFLICT OF INTEREST

The authors declare no conflict of interest.

## Data Availability

The data that support the findings of this study are available on request from the corresponding author. The data are not publicly available due to privacy or ethical restrictions.
